# LightBot: A Multi-Light Position Robotic Acquisition System for Adaptive Capturing of Cultural Heritage Surfaces

**DOI:** 10.3390/jimaging8050134

**Published:** 2022-05-12

**Authors:** Ramamoorthy Luxman, Yuly Emilia Castro, Hermine Chatoux, Marvin Nurit, Amalia Siatou, Gaëtan Le Goïc, Laura Brambilla, Christian Degrigny, Franck Marzani, Alamin Mansouri

**Affiliations:** 1 Laboratory of Imaging and Artificial Vision, Université de Bourgogne Franche-Compté, 21000 Dijon, France; yuly-emilia_castro-cartagena@etu.u-bourgogne.fr (Y.E.C.); hermine.chatoux@u-bourgogne.fr (H.C.); marvin.nurit@u-bourgogne.fr (M.N.); amalia.siatou@he-arc.ch (A.S.); gaetan.le-goic@u-bourgogne.fr (G.L.G.); franck.marzani@u-bourgogne.fr (F.M.); alamin.mansouri@u-bourgogne.fr (A.M.); 2 Haute Ecole Arc Conservation-Restauration, HES-SO, University of Applied Sciences and Arts Western Switzerland, 2000 Neuchâtel, Switzerland; laura.brambilla@he-arc.ch (L.B.); christian.degrigny@he-arc.ch (C.D.)

**Keywords:** reflectance transformation imaging (RTI), cultural heritage, robotic acquisition, RTI stitching

## Abstract

Multi-light acquisitions and modeling are well-studied techniques for characterizing surface geometry, widely used in the cultural heritage field. Current systems that are used to perform this kind of acquisition are mainly free-form or dome-based. Both of them have constraints in terms of reproducibility, limitations on the size of objects being acquired, speed, and portability. This paper presents a novel robotic arm-based system design, which we call LightBot, as well as its applications in reflectance transformation imaging (RTI) in particular. The proposed model alleviates some of the limitations observed in the case of free-form or dome-based systems. It allows the automation and reproducibility of one or a series of acquisitions adapting to a given surface in two-dimensional space.

## 1. Introduction

**Figure 1:**
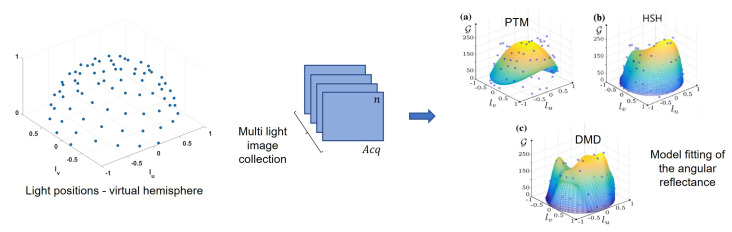
Reflectance transformation imaging technique. An example of the per pixel reflectance modeling using is shown here (**a**) PTM, (**b**) HSH and (**c**) DMD. These modelling plots are reprinted with permission from Pitard et al. [4].

Multi-light image collections regroup a series of techniques that allow the acquisition of a collection of images where only the lighting conditions vary (its spatial position or spectral content). We can cite, for instance, photometric stereo [1] and reflectance transformation imaging (RTI) [2], consisting of imaging an object from a fixed camera view while varying the light direction for each image captured. Photometric stereo has the goal of reconstructing a 3D view of the object, whereas RTI aims for interactive relighting of the surface. We focus on RTI systems as they have proven to provide an accurate description of important parameters related to the surface appearance and geometry and thus have found application in the study and analysis of cultural heritage (CH) surfaces [3]. Figure 1 illustrates the RTI process where images of a surface captured each with discrete lighting conditions are fitted to a mathematical model representing the reflectance of the surface.

**Figure 2:**
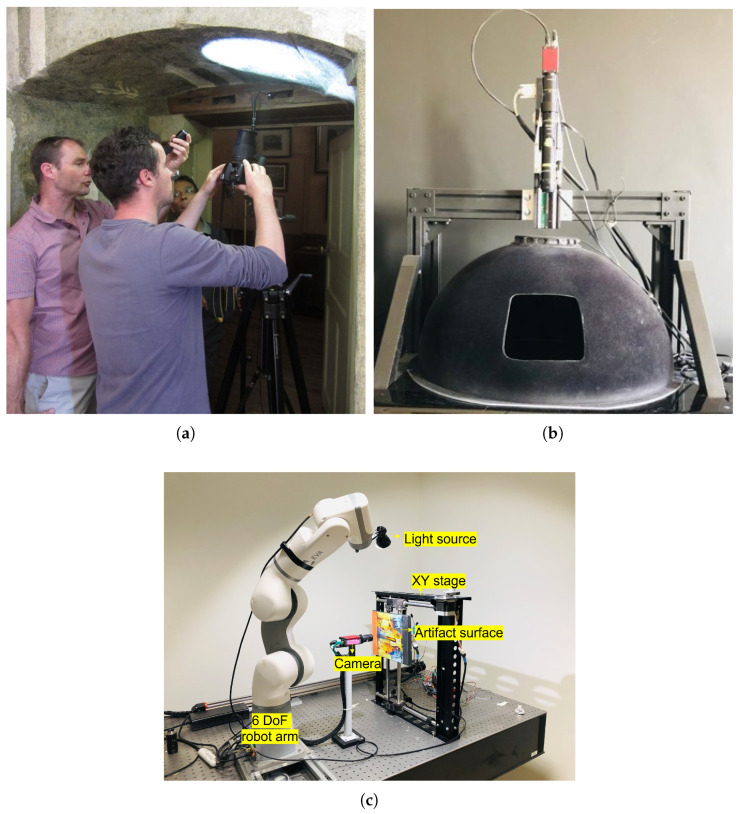
RTI acquisition systems. (**a**) Free-form highlight-RTI (a acquisition performed by our group), (**b**) Dome system [14], (**c**) Proposed system—LightBot.

The multi-light acquisition systems can be broadly categorized into two types: free-form highlight-RTI [5,6] or dome-based [7,8]. The free-form RTI systems provide more freedom in the setup since the acquisitions are usually performed with portable instrumentation using a handheld light source and a camera attached to a tripod with adjustable position. This allows multi-scale acquisitions; however, repeatability and reproducibility are compromised due to the difficulty of retrieving the same light positions. On the other hand, dome-based systems provide the necessary repeatability but have limitations on the object size as well as a limited angular area for light positioning and portability. In Figure 2, a typical free-form hightlight-RTI setup, a dome-based setup and our proposed system are shown for illustration.

LightBot is the first attempt to use both a robot arm to control the light position and an XY stage to control the surface position to enable several aspects of RTI, such as data stitching, adaptive acquisitions, etc. Kitanovski et al. [9] present a method using a robot arm for RTI acquisition, but the details of the system, its capabilities and limitations are not presented. Another robot-based system was designed and built by Santos et al. [10] for acquiring BRDF [11] data, where the robot arm is used to move the camera and a turntable is used to manipulate the object being scanned. In our case, the camera remains stationary and the robot arm is used to move the light source. This coupling can enable a lot of possibilities since it allows the creation of any type of virtual dome. This is an important feature, particularly for medium- and large-sized cultural heritage objects such as paintings, manuscripts, etc., having complex surfaces. Moreover, automation can find ground when there is a need to make repetitive acquisitions, such as in documenting a series of coin collections [12] or for monitoring the condition of an object over time [13]. The current state-of-the-art acquisition systems are unsuitable for large-scale surface measurement and stitching. The proposed system addresses this challenge of performing RTI on large-scale surfaces. We present the mechanical aspects of our system in Section 2. In Section 3, we demonstrate the capability and application of LightBot, showing the implementation and results of our methods on a few cultural heritage surfaces. In Section 4, we draw conclusions on the proposed system, discussing its applications and the plan for future systems.

## 2. Proposed System

LightBot, the system we propose, is an efficient, surface-adaptive RTI acquisition system designed and built for developing new methods in RTI. With the autonomous translation of the surface between acquisition sequences, it is, according to our knowledge, the first approach towards the automation of the RTI acquisition process for medium and large surfaces that cannot be acquired in the conventional dome systems. LightBot accepts the standard Light Positions (.lp) file containing a predefined set of light directions and executes the acquisition by moving the light source oriented in the given directions. Moreover, the system, when combined with the Next Best Light Position [15], can be used as a standalone unit to perform fully automated RTI acquisition adjustable to the complexity of the object’s surface geometry. Solving the inverse kinematics problem of the system and collision avoidance are key to trajectory planning while executing the acquisition.

The proposed system is limited by its cost and portability. On the other hand, the system addresses some of the important issues in the current RTI acquisition process, such as the time taken to perform acquisitions of a batch of surfaces in sequence with minimal intervention, stitching of RTI data, repeatability and reproducibility. A typical acquisition having 75 light positions takes around 4 min to complete using LightBot. The robot arm offers the flexibility to have different hemisphere sizes.

### 2.1. Engineering Design

The system comprises a collaborative robot arm, a light source attached to the end effector of the arm, a high-resolution camera and an XY positioning table for the precise translation of the object in the XY space with respect to the camera. Figure 2c shows the system setup that is used in this work. For the initial proof of concept, we used a 6 DoF lightweight robot arm—Automata Tech’s EVA robot arm. EVA stretches up to 600 mm in length, and the stroke length of each axis of the XY positioning table is 400 mm. This stroke length is ideal for translating surfaces ranging from a few cm to 50 cm for the current setup. This can be used to carry out RTI acquisitions of small- to medium-sized objects such as coins, printing plates, paintings, etc. We used Allied Vision’s Manta G504B camera (sensor size 2/3) with a suitable objective of the focal length of 3 mm to 6 mm to achieve an FoV of 150 mm width and 100 mm height at a working distance of 450 mm to 550 mm in our setup. However, in the next version of our setup, we use a longer and more dexterous robotic arm—the Kinova Jaco 7DoF—and a higher-resolution camera. In this new setup, we can have hemispheres of sizes ranging up to 90 cm in radii. Hemispheres larger than this are not necessary as, for such large surfaces, we must break the acquisition down into smaller parts and stitch them to maintain the resolution.

### 2.2. System Architecture

**Figure 3:**
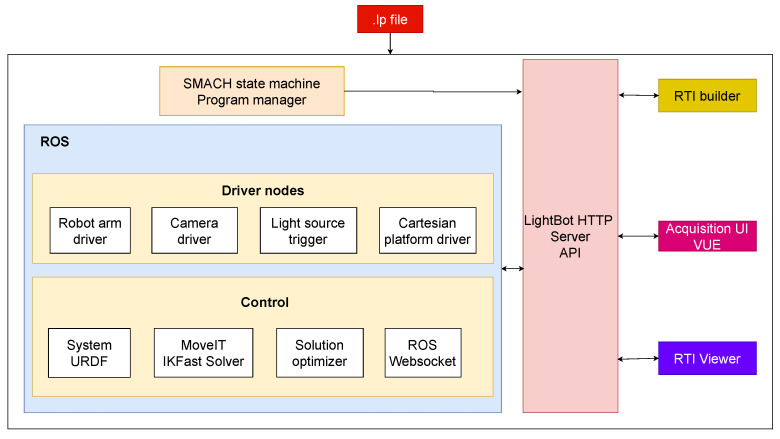
System architecture of the LightBot.

**Figure 4:**
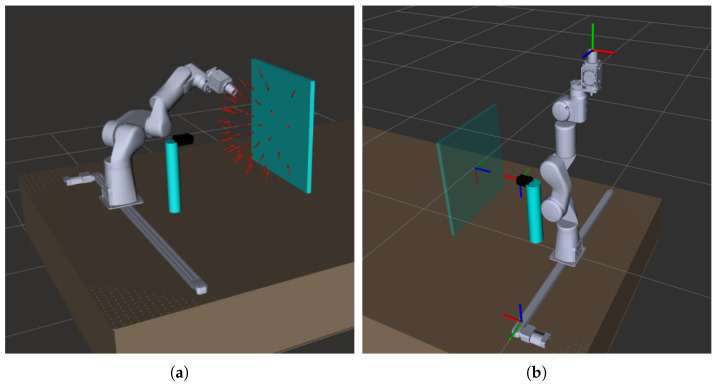
Visualization of the system in ROS RViz. (**a**) Visualization of the system and an example set of light poses in Rviz. (**b**) Reference frames to which the system components are attached.

Figure 3 represents an overview of the architecture of the LightBot system. The whole system is built on the ROS framework. In Figure 4a, the system as visualized in the RViz (ROS visualization tool) is shown. The red arrows in the figure represent the set of light poses in a sample acquisition. A HTTP server is built to control the system and an API documentation for the same is also developed. A simple-to-use web-based user interface is built using the VUE frontend framework with which the users (CH conservators) can carry out RTI acquisitions easily, having to simply upload the .lp file and set a few parameters, such as the surface size, etc.

### 2.3. Motion Planning

The light positions in the uploaded .lp file are transformed from the surface reference frame to the system world frame, which, in our case, is the arm base frame. All the transformations are carried out in the ROS frame transformation server. For a given target light position, a motion plan is generated using the rapidly exploring random tree star (RRT) method [16], which contains a sequence of robot arm trajectory points. Then, this sequence of points in the task space is converted to joint space for the robot to execute the motion. For every point in an identified valid trajectory path, there exists a list of inverse kinematics solutions. For computing the inverse kinematics of the robot, an IKFast plugin from the URDF of the robot arm was created using the IKFast kinematics solver in OpenRave. Each trajectory point in the task space is represented as a layer. Hence, for N trajectory points generated, there are N layers. The valid IK solutions in a layer are represented as nodes. Every node in an *i*th layer is connected to all the nodes in the (*i* + 1)th layer. The optimality of a path is represented by the travel cost from the start point to the endpoint in the joint space. The optimal path is chosen such that the sequence of nodes that connects a path from layer 1 to layer N has the lowest possible total cost defined in Equation (1).

(1) $$C_{i}=\sum_{i=0}^{N-2}{\left|p_{i}-p_{i+1}\right|}^{T}\cdot w,$$

where $\left\{\begin{array}{c} i\;\mathrm{represents}\;a\;\mathrm{layer},\\ p_{i}\;\mathrm{is}\;\mathrm{the}\;\mathrm{joint}\;\mathrm{position}\;\mathrm{in}\;\mathrm{layer}\;i,\\ w\;\mathrm{is}\;\mathrm{the}\;\mathrm{weights}\;\mathrm{assigned}\;\mathrm{to}\;\mathrm{individual}\;\mathrm{joints}. \end{array}\right.$

*w* is a 6 length vector determined empirically. It is the same for all positions $p_{i}$. For example, the base joint of the robot arm has the highest weight (the probability of collision is high), while the end-effector joint has the lowest weight (probability of hitting an object is almost null).

**Figure 5:**
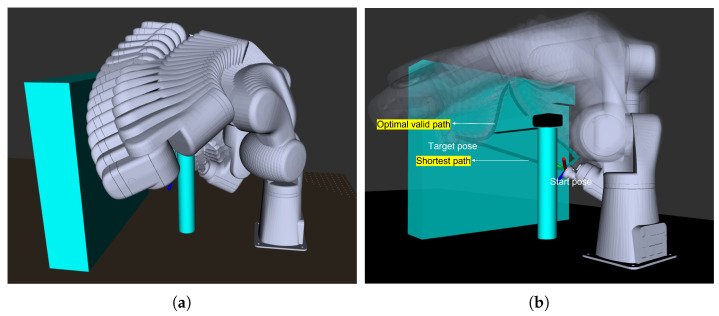
Planning of motion between two points with collision avoidance and optimal trajectory. (**a**) Planned sequence of the robot poses during its motion from the starting pose to the target pose. (**b**) Optimal valid path having the lowest cost vs. the shortest path.

Figure 5 illustrates the performance of the motion planning method implemented. In this example, the robot moves from a start pose to the target pose via the path that has the lowest cost and having no collisions with the objects in the environment. We use a PID controller to drive the robot joints smoothly while executing this optimal trajectory.

### 2.4. System Calibration

Currently, the system is set up on an optic table whereby the relative pose of each system component—robot arm, camera and XY platform—is fixed and known. However, self-calibration of the system in the absence of an optic table is done with the aid of fiducial markers. Hand–eye calibration [17] and the pose calibration between the camera frame of reference and XY platform frame of reference are the two calibrations involved in our system configuration. Hand–eye calibration is carried out by attaching the fiducial marker to the end-effector (light source). Calibration of the XY platform is carried out by attaching a fiducial marker to the XY platform within the camera FoV. The reference frames to which the system components are attached are shown in Figure 4b.

## 3. Applications

Museum custodians and conservators are still facing challenges in the digitization of the high-resolution geometry data of CH objects for both the documentation and analysis of the artifacts. The proposed system is focused on the efficient as well as scientifically reliable acquisition of RTI data. In this section, we demonstrate three such key applications—surface adaptive RTI acquisition with the adjustable radius of the light dome, RTI acquisition of medium and large surfaces using data stitching methods and automated batch processing for efficient large-scale RTI acquisitions. RTI was mainly created for the relighting of the surfaces and hence we have included only the relighted images to show the results.

### 3.1. Surface Adaptive Virtual Dome with Adjustable Radius

Conventional RTI acquisitions are performed with homogeneously distributed fixed light positions, regardless of the size and complexity of the surface being acquired. Luxman et al. [15] discuss the shortcomings of such acquisitions and their inadequacy in capturing the surface details. In order to fully realize the power of the RTI in revealing the surface phenomenon, it is important to choose the size of the virtual dome and the distribution of the light positions on it to be adaptive to the surface. LightBot carries the option of having a virtual dome of variable size. This opens up a lot of possibilities, and we believe that this will open up new perspectives for the automation of the RTI imaging process and the development of new methods towards surface-adaptive RTI acquisitions.

**Figure 6:**
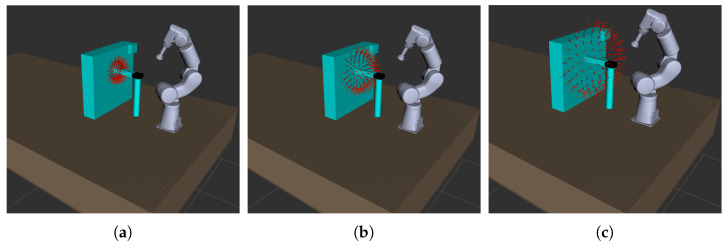
Dome configurations with variable radius. The radius of the dome can be adjusted from a few cm to 30 cm with this robot arm. (**a**) Hemisphere radius = 15 cm. (**b**) Hemisphere radius = 20 cm. (**c**) Hemisphere radius = 25 cm.

**Figure 7:**
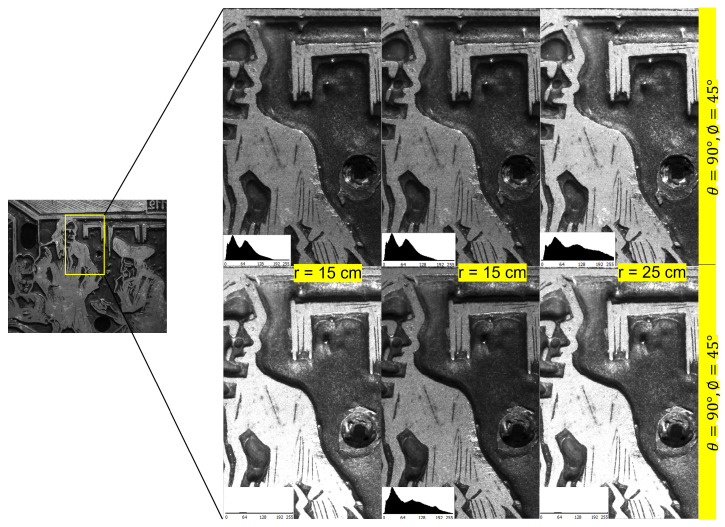
Relighting of the surface from RTI of the surface captured with dome of different sizes. In this example, the surface is relighted from $\theta =90^{\circ }$, $\phi =45^{\circ }$ and from $\theta =-90^{\circ }$, $\phi =45^{\circ }$.

Figure 6 illustrates the possibility of having different dome configurations in the proposed system. To illustrate the effect of the dome sizes on the quality of RTI, we made multiple acquisitions of a 20th century metal print plate using LightBot, having a dome radius of 15 cm, 20 cm and 25 cm, respectively. A sample of the outcome of these acquisitions is shown in Figure 7. It can be observed that the surface is more evenly illuminated in the acquisition made with a dome of size 25 cm compared to the other two when the surface is relighted from both position 1 and position 2. This uniformity of illumination directly affects the quality of the various maps extracted from the RTI data. It is interesting to note that the surface phenomenon is highlighted differently not only with the direction but also with the distance, and the behavior is not linear.

### 3.2. RTI of Large Surfaces Using Data Stitching Methods

**Figure 8:**
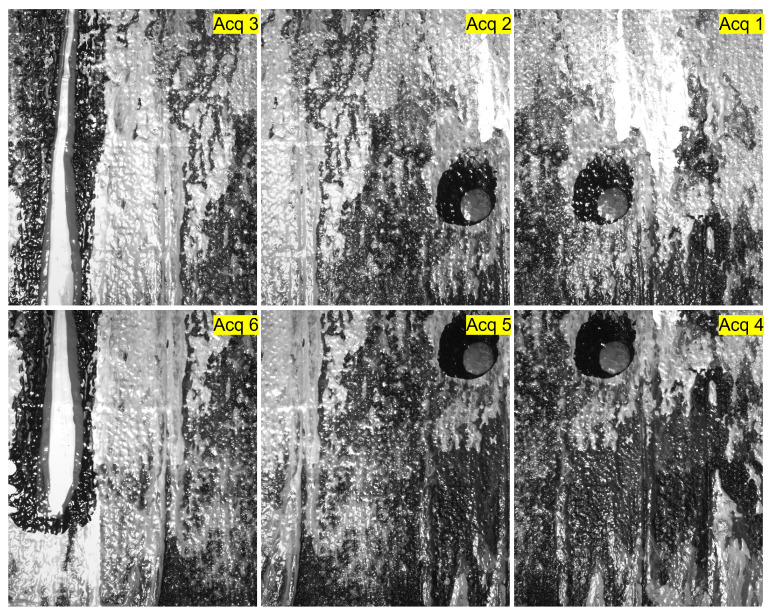
RTI acquisitions of a canvas painting (24.5 cm × 20 cm) in parts. There are in total 6 acquisitions, each covering an area of 9.3 cm × 7.6 cm with 30 % overlap between each pair of consecutive acquisitions.

**Figure 9:**
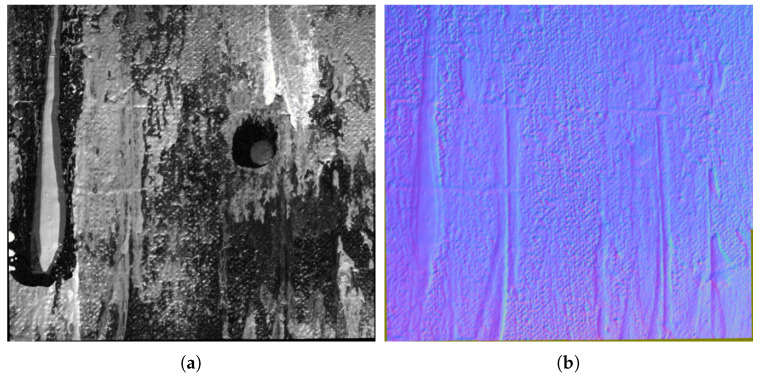
The acquired data are stitched to reconstruct the whole canvas painting. Visualization of the relighted image, normal map and directional slope obtained by processing of the RTI data using DMD [4] model fitting. (**a**) Relighted image. (**b**) Normal map.

To acquire images of large-scale surfaces without compromising the resolution, there is a need to break the acquisition down into multiple acquisitions, each capturing a smaller part of the surface. It is highly tedious to do this manually while ensuring the alignments. There are no existing systems for performing such acquisitions in the case of RTI. The proposed system addresses this problem. We demonstrate this capability of the system by performing the acquisition of a canvas painting. The acquisition is carried out in six parts, as shown in Figure 8. The surface is 24.5 cm long and 20 cm wide. It is translated along the X and Y directions by the XY positioning table, and RTI acquisitions with 75 homogeneously distributed light positions are performed at each translation. Each acquisition covers an area of 9.3 cm × 7.6 cm with an overlap of around 30% between consecutive regions. The data acquired are later stitched to perform RTI of the whole surface. The results of the stitching are illustrated in Figure 9, where a sample of the relighted surface and its normal map measured are shown. Stitching RTI data raises several issues and is, as such, a field of research under progress. In this example, we performed stitching using the illumination correction and registration method developed in-house. The details of the technique are beyond the scope of this paper. However, any illumination correction or flattening techniques such as [18,19] and registration methods as discussed in [20] can be used for performing the stitching depending on the surface being acquired.

### 3.3. Batch Acquisition

Another important rationale behind our proposed system design is the batch processing of the RTI acquisition workflow in large scale. This is a real need in museums, where manually performing the RTI acquisitions of so many artifacts is practically challenging and, even when performed, is prone to errors and is not reliable. To overcome this, we introduced a pipeline for the automated acquisition of multiple surfaces in batches, leveraging the degrees of freedom of the robot arm and the XY platform in LightBot.

**Figure 10:**
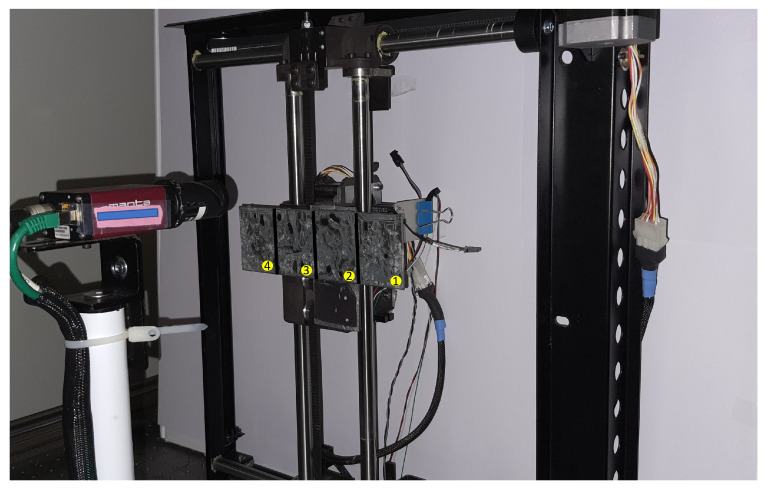
An example of batch acquisition where the system executes acquisition of 4 surfaces one after the other.

Figure 10 illustrates an example batch acquisition carried out where there are four surfaces attached to the XY stage. Acquisition of these four surfaces is carried out with minimal manual intervention by simply setting the initial position, final position and the intermediate steps along both the X and Y directions as the batch acquisition parameters in the LightBot WebAPI. Light positions can be configured either the same or differently for each acquisition depending on the surfaces being acquired. The system then executes the series of acquisitions, generating a new acquisition directory and the corresponding .lp file for each. Table 1 compares the acquisition time and number of human interventions required when the acquisition of these four surfaces is performed using the conventional RTI system and that using the LightBot system. Although the acquisition time per surface is almost the same, avoiding intervention between each surface acquisition effectively reduces the overall acquisition time. This simple yet powerful automation of the bulk acquisition saves a lot of time for the conservators and makes the acquisition process much easier and more reliable overall. The batch acquisition feature in LightBot is a direct offshoot of a specific need in museums as well as industries to perform RTI acquisition of metal coupons of sizes around 5 cm × 5 cm, as in the study of corrosion and change [21].

## 4. Conclusions

The LightBot is a proof of concept of a novel robot-based RTI system that is useful to perform automated multiple, repeatable acquisitions and to achieve surface shape/size-adaptive acquisitions. The human–machine interface of LightBot is simple and completely built over a HTTP webserver. Hence, with the API of the LightBot, which gives access to various functionalities of the system, it is convenient to develop new methods in RTI acquisition, such as NBLP [15], HD-RTI [22], data stitching, etc. The key benefits of this system design are demonstrated in this paper. The next version of LightBot is being built with an advanced and longer robotic arm, a high-resolution camera and a motor-actuated objective for auto-magnification and focus, along with an ROI finder using fiducial markers. Moreover, the new system is assembled having the robot arm and camera mounted top-down, enabling the placement of surfaces on a horizontal XY stage. With these additional features, the realization of a fully automated, surface-adaptive RTI acquisition system is envisaged.

## Figures and Tables

**Table 1:** Comparison of number of manual interventions and the acquisition time for acquiring 4 metal print plate samples using conventional system and that with the LightBot system.

	Acquisition Time	Number of Interventions
Conventional systems	$4\times T_{m}$	4
LightBot	$T_{b}\approx T_{m}$	1

## Data Availability

Not applicable.

## Abbreviations

The following abbreviations are used in this manuscript:

RTI	Reflectance Transformation Imaging
CH	Cultural Heritage
ROS	Robot Operating System
BRDF	Bidirectional Reflectance Distribution Function
H-RTI	Highlight Reflectance Transformation Imaging
LP	Light Positions
NBLP	Next Best Light Position
HD-RTI	High Dynamic range Reflectance Transformation Imaging
ROI	Region Of Interest
API	Application Programming Interface
